# Mol­ecular and crystal structure, Hirshfeld analysis and DFT investigation of 5-(furan-2-yl­methyl­idene)thia­zolo[3,4-*a*]benzimidazole-2-thione

**DOI:** 10.1107/S2056989020015017

**Published:** 2020-11-13

**Authors:** Hafsa Khaldi, Ahmed Djafri, Youcef Megrouss, Nawel Khelloul, Abdelkader Chouaih, Ayada Djafri

**Affiliations:** a Laboratory of Organic Applied Synthesis (LSOA), Department of Chemistry, Faculty of Sciences, University of Oran 1, Ahmed Ben Bella, 31000 Oran, Algeria; b Centre de Recherche Scientifique et Technique en Analyses Physico-Chimiques, (CRAPC), BP 384-Bou-Ismail-RP 42004, Tipaza, Algeria; c Laboratory of Technology and Solid Properties (LTPS), Abdelhamid Ibn Badis University, BP 227 Mostaganem 27000, Algeria

**Keywords:** crystal structure, benzimidazoles, thia­zoles, DFT analysis, reactivity

## Abstract

The thia­zolo[3,4-*a*]benzimidazole fused-ring system in the title compound, C_14_H_8_N_2_OS_2_, is nearly planar, the r.m.s. deviation being 0.0073 Å. The thia­zolo-benzimidazole-2-thione system is almost in the same plane as the furan-2-yl-methyl­ene moiety, with a dihedral angle of 5.6 (2)° between the two least-squares planes. In the crystal, adjacent mol­ecules are connected by weak inter­molecular inter­actions (C—H⋯N and slipped π–π stacking) into a three-dimensional network. The nature of the inter­molecular inter­actions was also qu­anti­fied by Hirshfeld surface analysis. DFT analysis indicates a good agreement of the experimentally determined and the theoretically calculated mol­ecular structures.

## Abstract 2

The crystal structure of the title compound is stabilized by the presence of weak C—H⋯N hydrogen bonds and slipped π–π inter­actions. Hirshfeld surface analysis showed that H⋯H contacts are the dominant inter­actions.

## Chemical context   

The synthesis and biological activity of thia­zolo­benz­imid­azoles were first studied several decades ago (Ogura *et al.*, 1968 ▸; Krasovskii & Kochergin, 1972 ▸; Alper & Taurins, 1967 ▸). With regard to their biological activity, thia­zolobenzimidazole derivatives have been evaluated in particular for their inhibitory effects on HIV-1 (Chimirri *et al.*, 1999 ▸; Roth *et al.*, 1997 ▸) and their use as anti­bacterial (Oh *et al.*, 1995 ▸), anti-inflammatory (Bender *et al.*, 1985 ▸), anti­diabetic (El-Shorbagi *et al.*, 2001 ▸), broncholytic (Park *et al.*, 1993 ▸), anti­protozoal (Singh, 1970 ▸), anti­convulsant (Sharpe *et al.*, 1971 ▸) and anti­depressant (Miller & Bambury, 1972 ▸) agents. Some thia­zolo­benz­imid­azole derivatives are also used for the treatment of cancer and bone diseases (Al-Rashood & Abdel-Aziz, 2010 ▸). Furthermore, compounds with the benzimidazole moiety have been developed into useful materials for usage in non-linear optical fields (Vijayan *et al.*, 2004 ▸) or photovoltaic cells (Bodedla *et al.*, 2016 ▸; Gong *et al.*, 2010 ▸).
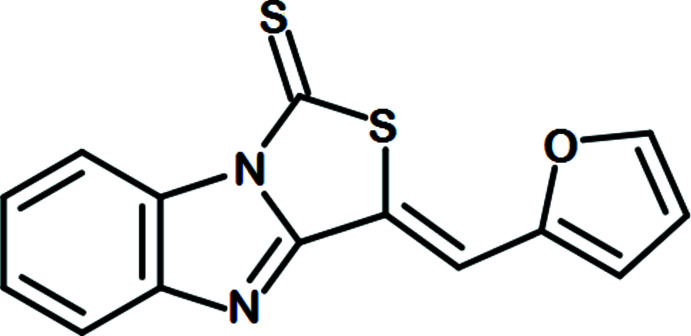


We report in this communication the synthesis, mol­ecular and crystal structures and Hirshfeld surface analysis of the title thia­zolo derivative. In addition, the HOMO–LUMO energies, mol­ecular electrostatic potential and chemical reactivity descriptors are described on the basis of theoretical calculations.

## Structural commentary   

**Figure 1:**
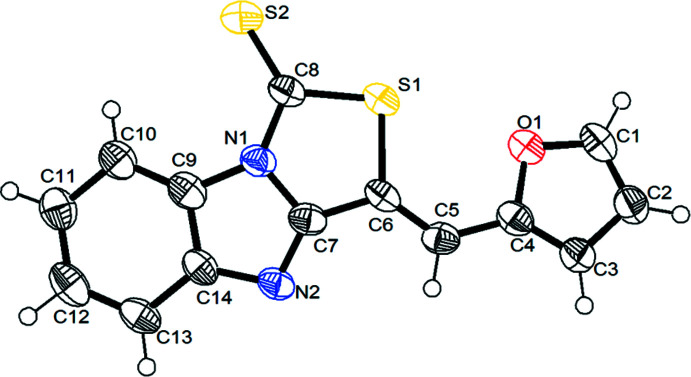
The mol­ecular structure of the title compound showing the atom-numbering scheme. Displacement ellipsoids are drawn at the 50% probability level.

The mol­ecular structure of the title compound is shown in Fig. 1 ▸. The tricyclic thia­zolobenzimidazole group, consisting of a benzimidazole unit fused to a thia­zole ring, is bonded to a furan-2-yl-methyl­ene moiety at carbon atom C6. As expected, the thia­zolo[3,4-*a*]benzimidazole group is planar with an r.m.s. deviation of 0.0073 Å for the thirteen (C6–C14/N1/N2/S1/S2) non H-atoms. The furan-2-yl-methyl­ene moiety is also planar, with an r.m.s deviation of 0.0028 Å for the six (C1–C5/O1) non H-atoms. The two ring systems are almost in the same plane, their least-squares planes subtending a dihedral angle of 5.6 (2)°. The mol­ecule exists in a *Z* configuration with respect to the C5=C6 bond. The S1—C8 and S1—C6 distances, 1.739 (4) and 1.775 (3) Å, respectively, are in agreement with a C—S single bond of a thia­zole ring (Rahmani *et al.*, 2016 ▸). In comparison, the S2—C8 bond [1.612 (4) Å] of the thione moiety is much shorter as a result of its double-bond character and the presence of a delocalized π-electronic system throughout the entire thia­zolo­benz­imid­azole ring system (Liang *et al.*, 2009 ▸). The bond lengths of the thia­zolobenzimidazole and furan rings are similar than those in a series of thia­zolo[3,2-*a*]benzimidazole and thia­zolo[3,4-*a*]benzimidazole compounds (Bruno *et al.*, 1996 ▸; Wang *et al.*, 2011 ▸). The intra­molecular C10—H10⋯S2 hydrogen-bonding inter­action (Table 1 ▸) helps to stabilize the mol­ecular conformation.

## Supra­molecular features and Hirshfeld surface analysis   

**Figure 2:**
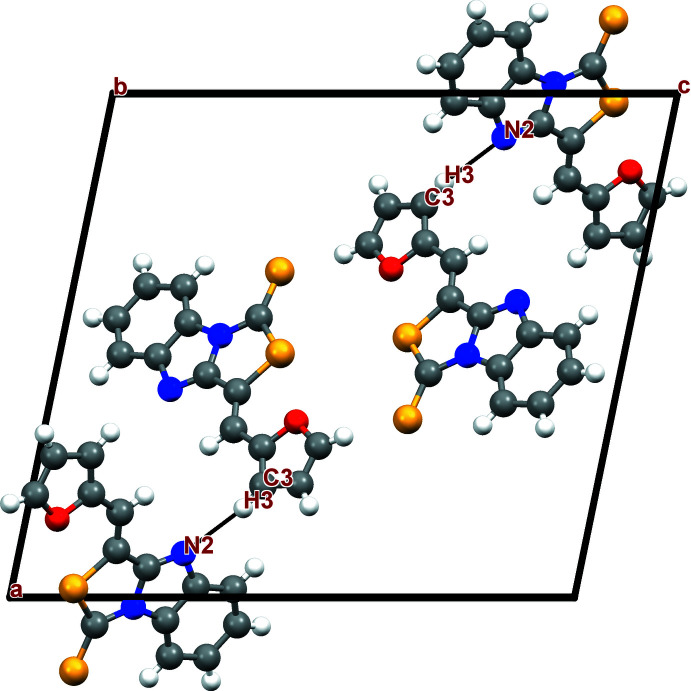
Crystal packing diagram of the title compound with hydrogen bonds (dashed lines) viewed along the *b* axis.

In similar reported structures containing thia­zole ring systems, the crystal packing is mainly based on short contacts and weak π–π inter­actions (Djafri *et al.*, 2017 ▸). In the crystal packing of the title compound, weak C3—H3_aromatic_⋯N2^i^ hydrogen bonds (Table 1 ▸) connect the mol­ecules into dimers (Fig. 2 ▸). Additional π–π stacking inter­actions between adjacent thia­zolobenzimidazole ring systems link the dimers into a three-dimensional network structure, with centroid-to-centroid distances of 3.6523 (18) Å (slippage 1.141 Å) and 3.6515 (1) Å (slippage 1.137 Å) between the thia­zole ring and the benzene ring of one thia­zolobenzimidazole ring system, and between the imidazole ring and the benzene ring of another thia­zolo­benz­imid­azole ring system, respectively.

**Figure 3:**
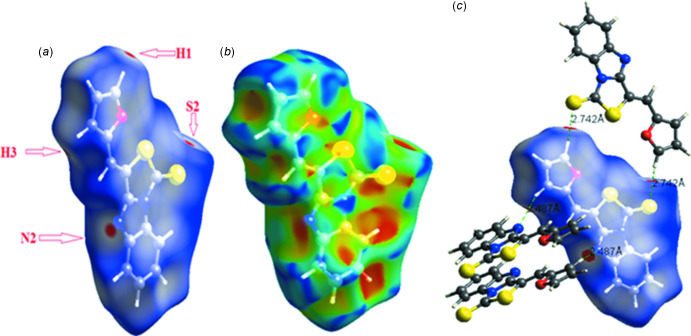
Hirshfeld surfaces for visualizing the inter­molecular contacts of the title compound: (*a*) *d*_norm_ Hirshfeld surface, (*b*) shape-index and (*c*) *d*_norm_ highlighting the regions of the C—H⋯S and C—H⋯N hydrogen bonds.

**Figure 4:**
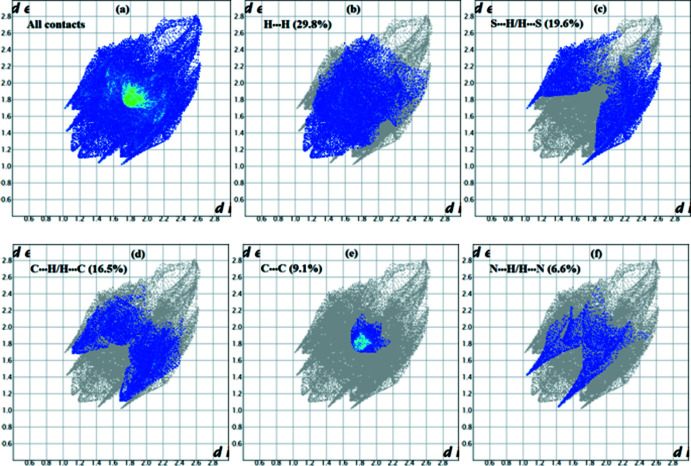
Two-dimensional fingerprint plots showing the contributions of different types of inter­actions: (*a*) all inter­molecular contacts, (*b*) H⋯H contacts, (*c*) S⋯H/H⋯S contacts, (*d*) C⋯H/H⋯C contacts, (*e*) C⋯C contacts and (*f*) N⋯H/H⋯N contacts.

Hirshfeld surface (HS) analysis of the title compound was performed using *Crystal Explorer 17* (Turner *et al.*, 2017 ▸) with the surface mapped over *d*_norm_ as described in the literature (Yahiaoui *et al.*, 2019 ▸). In the *d*_norm_ surface, strong inter­molecular inter­actions appear as red spots (Bahoussi *et al.*, 2017 ▸; Khelloul *et al.*, 2016 ▸) as depicted in Fig. 3 ▸ *a* (here origin­ating particularly from the C—H⋯N hydrogen bond). The presence of π–π stacking inter­actions is indicated by red and blue triangles on the shape-index surface as can be seen in Fig. 3 ▸ *b*. In Fig. 3 ▸ *c*, the other red spots indicate also the presence of a weaker C—H⋯S hydrogen bond (between H3 and S2) and C—H⋯N (between H1 and N2). The overall two-dimensional fingerprint (FP) plots, and those delineated into H⋯H, C⋯H/H⋯C, S⋯H/H⋯S, N⋯H/H⋯N and C⋯C contacts are shown in Fig. 4 ▸. H⋯H contacts are the dominant inter­actions with a contribution of 29.8% to the overall HS. The S⋯H/H⋯S inter­actions appear as the next largest region of the FP plot, highly concentrated at the edges, characteristic of hydrogen-bond inter­actions with an overall HS contribution of 19.6%. The C⋯H/H⋯C inter­actions are illustrated by two symmetrical wings on the left and right sides (16.5% contribution). The C⋯C contacts, which are the measure of π–π stacking inter­actions, occupy 9.1% of the HS and appear as a unique triangle. The N⋯H/H⋯N contacts are represented by a pair of sharp spikes and make a contribution of 6.6%. Other inter­molecular contacts in the HS mapping contribution less than 5%.

## Theoretical calculations   

The hybrid functional B3LYP (Becke’s three-parameter hybrid model using the Lee-Yang Parr correlation functional) with the 6-311G (d, p) basis set (Becke, 1993 ▸) were used in all calculations as implemented in *Gaussian 09* (Frisch *et al.*, 2009 ▸). Theoretical calculations were performed to obtain the optimized mol­ecular structure of the title compound in the gas phase. The crystallographic information file was used as an input file in the *GaussView 5* program (Frisch *et al.*, 2000 ▸) to start structure optimization of the title compound. Comparison of the DFT-optimized mol­ecular structure with the refined structure based on single crystal X-ray data revealed a good agreement (see supporting information for a detailed comparison of bond lengths and angles). Frontier mol­ecular orbitals and the mol­ecular electrostatic potential were calculated using the same level of theory.

## Frontier mol­ecular orbital and chemical reactivity   

**Figure 5:**
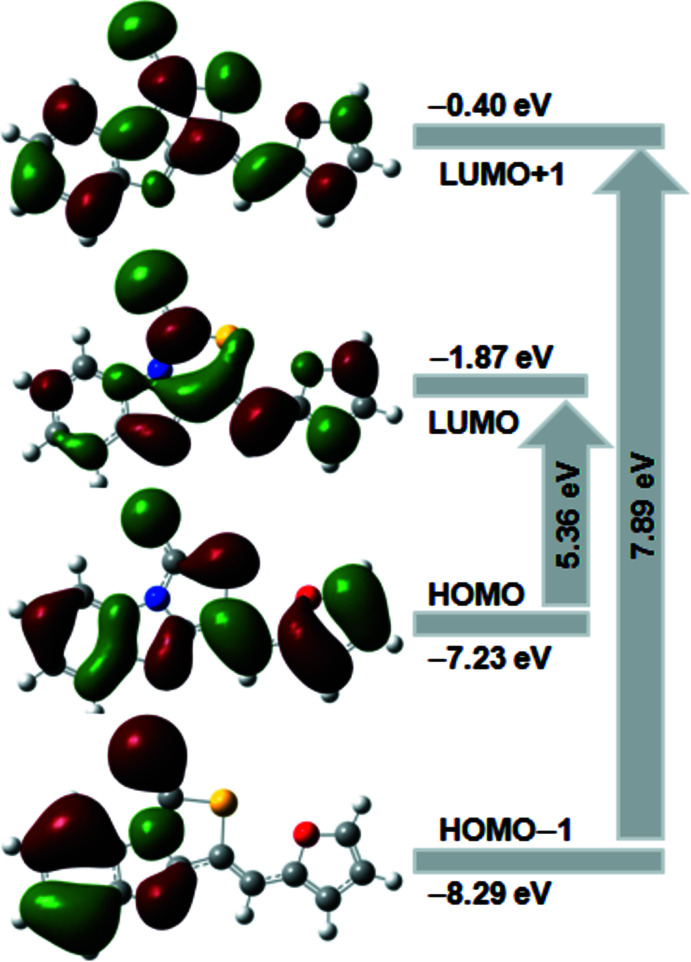
Mol­ecular orbitals plot showing the frontier orbitals.

The frontier mol­ecular orbitals, HOMO (highest occupied mol­ecular orbital) and LUMO (lowest-unoccupied mol­ecular orbital), are plotted to specify the distribution of electronic densities. The electron distribution of the HOMO-1, HOMO, LUMO and the LUMO+1 energy levels are shown in Fig. 5 ▸. As can be seen from the figure, the HOMO and LUMO are localized in the plane extending from the whole furan ring to the thia­zolo-benzimidazole ring system. The frontier mol­ecular orbital energies, EHOMO and ELUMO are −7.23 and −1.87 eV, respectively, and the HOMO–LUMO gap is 5.36 eV. Since the gap energy is considered to be small, the mol­ecule is defined as soft.

Global chemical reactivity descriptor (GCRD) parameters can be obtained as reported in the literature (Belkafouf *et al.*, 2019 ▸). The calculated values of the GCRD parameters for the title mol­ecule are summarized in Table 2 ▸. The chemical stability of the title mol­ecule is explained by the chemical potential (μ) value, which is −4.55 eV. On the other hand, the chemical hardness (η) value is 2.68 eV, indicating that the charge transfer occurs within the mol­ecule. From Table 2 ▸, the electrophilic behaviour of the mol­ecule is confirmed by the global electrophilicity (ω), which has a value of 3.86 eV. The structure–property relationship can be also described by the hyper-hardness descriptor (Γ), which was introduced to investigate the reactivity or stability of mol­ecules theoretically (Ghanavatkar *et al.*, 2020 ▸). According to the results, the positive value of Γ (+4.30 eV) indicates stability of the mol­ecule.

## Mol­ecular electrostatic potential analysis   

**Figure 6:**
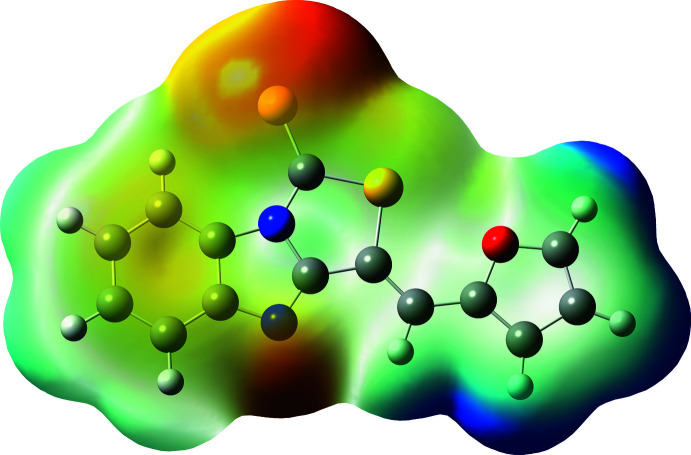
Mol­ecular electrostatic potential map of the title mol­ecule.

To predict reactive sites for electrophilic and nucleophilic attack, mol­ecular electrostatic potential (MEP) surfaces were computed at the B3LYP/6-311G (d,p) level with the optimized structure using *GaussView* (Frisch *et al.*, 2000 ▸). The different values of the electrostatic potential at the MEP surface are represented by red, blue and green (Kourat *et al.*, 2020 ▸). From Fig. 6 ▸, it is obvious that the negative potential regions (red) are associated with sulfur and nitro­gen atoms whereas the positive potential regions (blue) are on the side of hydrogen atoms. It may also be seen in Fig. 6 ▸ that green areas cover parts of the mol­ecule enveloping the π system of the aromatic rings.

## Synthesis and spectral characterization   

**Figure 7:**
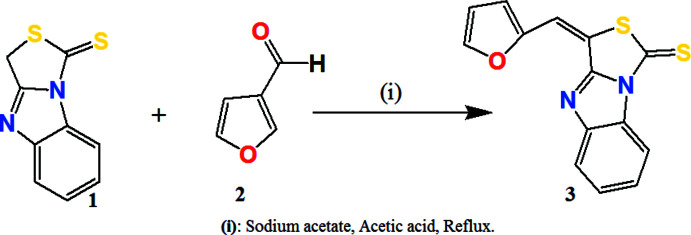
Synthetic route for the title compound (**3**).

The synthetic route preparation of the title compound is illustrated in Fig. 7 ▸. Initially, the tricyclic thia­zolo(3,4-*a*)benzimidazole (**1**) was obtained from amino phenyl­ene di­thio­carbamate and chloro­acetic acid by the Hanztsch reaction. The title compound (**3**) was prepared by Knoevenagel condensation of furaldehyde **2** (2; 0.01 mol) and the tricyclic compound (**1**; 0.02 mol) in acetic acid (10 ml) buffered by sodium acetate (0.02 mol). The reaction was monitored by TLC (petroleum ether/ethyl acetate, 8/2). After 4 h of refluxing and stirring, the brown solid obtained was filtered off, dried and recrystallized from ethanol to give the title compound, m.p. 493 K, in a yield of 85%.

Spectroscopic data (FT–IR, ^1^H NMR and ^13^C NMR) for (**3**). IR (KBr, cm^−1^): 3099, 3076 and 3026 (C*sp* ^2^—H_arom_), 1602 (C=N), 1555–1464 (C=C), 1390 (C=S), 1324 (–C—S–), 1259 (C—N) and 815, 759 (C—H_ar­yl_). ^1^H NMR (300 MHz, CDCl_3_, δ ppm) *J* (Hz): 6,6 (*q*, 1H, *J*_3_ = 1.76 Hz, furan), 6.80 (*d*, 1H, *J* = 3.48 Hz, furan), 7.45 (*m*, 2H, *J*_3_ = 1.66 Hz, *J*_4_ = 5,60 phen­yl), 7.60 (*s*, 1H, C=CH), 7.70 (*s*, 1H, furan) , 7.80 (*d*, 1H, *J*_3_ = 8.56 Hz, phen­yl), 8.50 (*d*, 1H, *J*_3_ = 8.72 Hz, phen­yl). ^13^C NMR (75 MHz, CDCl_3_, δ ppm): 113.46, 113.51, 113.90, 117.15, 118.81, 120.65, 121.32, 125.59, 126.55, 131.00, 146.63, 149.48, 150.42 (C=N), 187.15 (C=S).

## Refinement   

Crystal data, data collection and structure refinement details are summarized in Table 3 ▸. H atoms were placed in calculated positions (C—H = 0.93 Å) and allowed to ride on their parent atoms with *U*_iso_(H) = 1.2*U*_eq_(C).

## Supplementary Material

Crystal structure: contains datablock(s) I. DOI: 10.1107/S2056989020015017/wm5587sup1.cif

Structure factors: contains datablock(s) I. DOI: 10.1107/S2056989020015017/wm5587Isup2.hkl

Supporting information file. DOI: 10.1107/S2056989020015017/wm5587Isup3.mol

Selected bond lengths, bond angles and torsion angles for the non-hydrogen atoms, as determined by X-ray diffraction technique and DFT calculations. DOI: 10.1107/S2056989020015017/wm5587sup5.docx

Supporting information file. DOI: 10.1107/S2056989020015017/wm5587Isup5.cml

CCDC reference: 2043709

Additional supporting information:  crystallographic information; 3D view; checkCIF report

## Figures and Tables

**Table 1:** Hydrogen-bond geometry (Å, °)

*D*—H⋯*A*	*D*—H	H⋯*A*	*D*⋯*A*	*D*—H⋯*A*
C10—H10⋯S2	0.93	2.94	3.476 (4)	119
C3—H3⋯N2^i^	0.93	2.62	3.474 (5)	154
C1—H1⋯S2^ii^	0.93	2.89	3.788 (4)	163

Symmetry codes: (i) 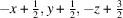; (ii) 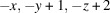.

**Table 2:** Frontier mol­ecular orbital energies (eV) and global chemical reactivity descriptors calculated using B3LYP/6–311G(d,p) level of theory

Parameter	Calculated energy
EHOMO	−7.23
ELUMO	−1.87
EHOMO−1	−8.29
ELUMO+1	−0.40
EHOMO–ELUMO (gap)	5.36
EHOMO−1 − ELUMO+1 (gap)	7.89
Ionization potential (*I*)	7.23
Electron affinity (*A*)	1.87
Chemical hardness (η)	2.68
Chemical potential (μ)	–4.55
Electronegativity (χ)	4.55
Electrophilicity (ω)	3.86
Hyper-hardness (Γ)	4.30

**Table 3:** Experimental details

Crystal data	
Chemical formula	C_14_H_8_N_2_OS_2_
*M*_r_	284.34
Crystal system, space group	Monoclinic, *P*2_1_/*n*
Temperature (K)	295
*a*, *b*, *c* (Å)	15.768 (5), 4.7583 (15), 17.316 (6)
β (°)	101.572 (8)
*V* (Å^3^)	1272.8 (7)
*Z*	4
Radiation type	Mo *K*α
μ (mm^−1^)	0.41
Crystal size (mm)	0.58 × 0.21 × 0.20

Data collection	
Diffractometer	Nonius Kappa CCD
Absorption correction	Multi-scan (Blessing, 1995 ▸)
*T*_min_, *T*_max_	0.856, 0.919
No. of measured, independent and observed [*I* > 2σ(*I*)] reflections	10879, 2281, 1835
*R*_int_	0.020
(sin θ/λ)_max_ (Å^−1^)	0.602

Refinement	
*R*[*F* ^2^ > 2σ(*F* ^2^)], *wR*(*F* ^2^), *S*	0.052, 0.113, 0.96
No. of reflections	2281
No. of parameters	172
H-atom treatment	H-atom parameters constrained
Δρ_max_, Δρ_min_ (e Å^−3^)	0.17, −0.17

Computer programs: *KappaCCD* (Nonius, 1998 ▸), *DENZO* and *SCALEPACK* (Otwinowski & Minor, 1997 ▸), *SHELXS97* (Sheldrick, 2008 ▸), *ORTEP-3 for Windows* (Farrugia, 2012 ▸), *Mercury* (Macrae *et al.*, 2020 ▸), *SHELXL2014* (Sheldrick, 2015 ▸), *PLATON* (Spek, 2020 ▸) and *publCIF* (Westrip, 2010 ▸).

## supplementary crystallographic information

### Crystal data

**Table:**

C_14_H_8_N_2_OS_2_	*F*(000) = 584
*M**_r_* = 284.34	*D*_x_ = 1.484 Mg m^−^^3^
Monoclinic, *P*2_1_/*n*	Mo *K*α radiation, λ = 0.71073 Å
*a* = 15.768 (5) Å	Cell parameters from 100 reflections
*b* = 4.7583 (15) Å	θ = 2.0–25.3°
*c* = 17.316 (6) Å	µ = 0.41 mm^−^^1^
β = 101.572 (8)°	*T* = 295 K
*V* = 1272.8 (7) Å^3^	Prism, colourless
*Z* = 4	0.58 × 0.21 × 0.20 mm

### Data collection

**Table:**

Nonius Kappa CCD diffractometer	1835 reflections with *I* > 2σ(*I*)
Radiation source: sealed tube	*R*_int_ = 0.020
θ/2θ scans	θ_max_ = 25.4°, θ_min_ = 3.6°
Absorption correction: multi-scan (Blessing, 1995)	*h* = −18→18
*T*_min_ = 0.856, *T*_max_ = 0.919	*k* = −5→5
10879 measured reflections	*l* = −20→20
2281 independent reflections	

### Refinement

**Table:**

Refinement on *F*^2^	0 restraints
Least-squares matrix: full	Hydrogen site location: inferred from neighbouring sites
*R*[*F*^2^ > 2σ(*F*^2^)] = 0.052	H-atom parameters constrained
*wR*(*F*^2^) = 0.113	*w* = 1/[σ^2^(*F*_o_^2^) + (0.0089*P*)^2^ + 4.0499*P*] where *P* = (*F*_o_^2^ + 2*F*_c_^2^)/3
*S* = 0.96	(Δ/σ)_max_ < 0.001
2281 reflections	Δρ_max_ = 0.17 e Å^−^^3^
172 parameters	Δρ_min_ = −0.17 e Å^−^^3^

### Special details

**Table:**

Geometry. All esds (except the esd in the dihedral angle between two l.s. planes) are estimated using the full covariance matrix. The cell esds are taken into account individually in the estimation of esds in distances, angles and torsion angles; correlations between esds in cell parameters are only used when they are defined by crystal symmetry. An approximate (isotropic) treatment of cell esds is used for estimating esds involving l.s. planes.

### Fractional atomic coordinates and isotropic or equivalent isotropic displacement parameters (Å^2^)

**Table:**

	*x*	*y*	*z*	*U*_iso_*/*U*_eq_	
S1	0.01611 (6)	0.1179 (2)	0.89016 (5)	0.0504 (3)	
S2	−0.14717 (8)	−0.2152 (3)	0.85975 (7)	0.0732 (4)	
O1	0.15244 (16)	0.5043 (6)	0.94349 (14)	0.0523 (7)	
C14	0.0218 (2)	−0.4142 (8)	0.67488 (18)	0.0432 (9)	
N1	−0.01982 (18)	−0.2326 (7)	0.77765 (15)	0.0436 (7)	
C9	−0.0453 (2)	−0.4245 (9)	0.71658 (19)	0.0467 (9)	
C7	0.0587 (2)	−0.1218 (8)	0.76838 (19)	0.0427 (9)	
C11	−0.1192 (3)	−0.7634 (9)	0.6302 (2)	0.0536 (10)	
H11	−0.1661	−0.8825	0.6139	0.064*	
N2	0.08686 (18)	−0.2264 (7)	0.70874 (15)	0.0430 (7)	
C8	−0.0552 (2)	−0.1294 (9)	0.83946 (19)	0.0467 (9)	
C13	0.0170 (2)	−0.5792 (9)	0.60819 (19)	0.0485 (10)	
H13	0.0595	−0.5718	0.5780	0.058*	
C6	0.0923 (2)	0.0822 (8)	0.82795 (19)	0.0430 (9)	
C5	0.1667 (2)	0.2165 (8)	0.8349 (2)	0.0433 (9)	
H5	0.1996	0.1713	0.7976	0.052*	
C3	0.2789 (2)	0.5589 (8)	0.9074 (2)	0.0490 (9)	
H3	0.3240	0.5411	0.8804	0.059*	
C10	−0.1160 (2)	−0.5960 (9)	0.6964 (2)	0.0503 (10)	
H10	−0.1595	−0.6002	0.7255	0.060*	
C4	0.2034 (2)	0.4222 (8)	0.8920 (2)	0.0462 (9)	
C1	0.2005 (3)	0.6951 (9)	0.9914 (2)	0.0551 (10)	
H1	0.1822	0.7867	1.0326	0.066*	
C2	0.2770 (3)	0.7349 (9)	0.9723 (2)	0.0540 (10)	
H2	0.3206	0.8552	0.9969	0.065*	
C12	−0.0534 (3)	−0.7544 (9)	0.5885 (2)	0.0574 (11)	
H12	−0.0568	−0.8722	0.5452	0.069*	

### Atomic displacement parameters (Å^2^)

**Table:**

	*U*^11^	*U*^22^	*U*^33^	*U*^12^	*U*^13^	*U*^23^
S1	0.0569 (6)	0.0590 (6)	0.0373 (5)	−0.0007 (5)	0.0142 (4)	−0.0071 (5)
S2	0.0644 (7)	0.0958 (10)	0.0690 (7)	−0.0167 (7)	0.0360 (6)	−0.0209 (7)
O1	0.0584 (16)	0.0575 (17)	0.0423 (14)	−0.0015 (14)	0.0132 (12)	−0.0078 (13)
C14	0.053 (2)	0.047 (2)	0.0274 (16)	0.0062 (19)	0.0031 (15)	0.0015 (17)
N1	0.0464 (17)	0.0520 (19)	0.0331 (15)	0.0008 (15)	0.0099 (13)	0.0005 (14)
C9	0.052 (2)	0.052 (2)	0.0342 (18)	0.0062 (19)	0.0032 (16)	0.0032 (18)
C7	0.048 (2)	0.050 (2)	0.0316 (17)	0.0049 (18)	0.0099 (15)	0.0058 (17)
C11	0.058 (2)	0.058 (3)	0.040 (2)	0.000 (2)	−0.0011 (17)	−0.003 (2)
N2	0.0497 (17)	0.0521 (19)	0.0271 (14)	0.0028 (15)	0.0079 (13)	0.0038 (14)
C8	0.052 (2)	0.056 (2)	0.0347 (18)	0.0025 (19)	0.0132 (16)	−0.0015 (18)
C13	0.060 (2)	0.056 (3)	0.0300 (17)	0.009 (2)	0.0082 (16)	0.0036 (18)
C6	0.049 (2)	0.049 (2)	0.0316 (17)	0.0033 (19)	0.0103 (15)	−0.0033 (17)
C5	0.054 (2)	0.040 (2)	0.0389 (18)	0.0017 (18)	0.0157 (16)	0.0063 (17)
C3	0.055 (2)	0.053 (3)	0.0399 (19)	−0.003 (2)	0.0102 (17)	−0.0020 (18)
C10	0.057 (2)	0.058 (3)	0.0334 (18)	0.004 (2)	0.0020 (16)	0.0011 (19)
C4	0.055 (2)	0.046 (2)	0.0377 (19)	0.0021 (19)	0.0106 (16)	0.0023 (18)
C1	0.069 (3)	0.055 (3)	0.041 (2)	0.003 (2)	0.0097 (19)	−0.010 (2)
C2	0.061 (2)	0.057 (3)	0.041 (2)	−0.006 (2)	0.0041 (18)	−0.006 (2)
C12	0.072 (3)	0.059 (3)	0.037 (2)	0.013 (2)	−0.0003 (19)	−0.008 (2)

### Geometric parameters (Å, º)

**Table:**

S1—C8	1.739 (4)	C11—C10	1.388 (5)
S1—C6	1.775 (3)	C11—H11	0.9300
S2—C8	1.612 (4)	C13—C12	1.376 (6)
O1—C1	1.354 (5)	C13—H13	0.9300
O1—C4	1.372 (4)	C6—C5	1.320 (5)
C14—C13	1.386 (5)	C5—C4	1.429 (5)
C14—C9	1.397 (5)	C5—H5	0.9300
C14—N2	1.397 (5)	C3—C4	1.336 (5)
N1—C7	1.384 (4)	C3—C2	1.407 (5)
N1—C8	1.392 (4)	C3—H3	0.9300
N1—C9	1.394 (5)	C10—H10	0.9300
C9—C10	1.368 (5)	C1—C2	1.326 (5)
C7—N2	1.302 (4)	C1—H1	0.9300
C7—C6	1.438 (5)	C2—H2	0.9300
C11—C12	1.378 (5)	C12—H12	0.9300
			
C8—S1—C6	94.40 (17)	C5—C6—C7	125.8 (3)
C1—O1—C4	105.1 (3)	C5—C6—S1	126.6 (3)
C13—C14—C9	119.3 (4)	C7—C6—S1	107.6 (3)
C13—C14—N2	128.7 (3)	C6—C5—C4	128.6 (3)
C9—C14—N2	112.0 (3)	C6—C5—H5	115.7
C7—N1—C8	117.4 (3)	C4—C5—H5	115.7
C7—N1—C9	106.9 (3)	C4—C3—C2	106.7 (3)
C8—N1—C9	135.7 (3)	C4—C3—H3	126.6
C10—C9—N1	132.9 (3)	C2—C3—H3	126.6
C10—C9—C14	123.5 (3)	C9—C10—C11	116.5 (4)
N1—C9—C14	103.6 (3)	C9—C10—H10	121.8
N2—C7—N1	113.7 (3)	C11—C10—H10	121.8
N2—C7—C6	133.7 (3)	C3—C4—O1	110.3 (3)
N1—C7—C6	112.6 (3)	C3—C4—C5	133.8 (3)
C12—C11—C10	120.5 (4)	O1—C4—C5	115.9 (3)
C12—C11—H11	119.7	C2—C1—O1	111.6 (3)
C10—C11—H11	119.7	C2—C1—H1	124.2
C7—N2—C14	103.8 (3)	O1—C1—H1	124.2
N1—C8—S2	126.5 (3)	C1—C2—C3	106.3 (4)
N1—C8—S1	108.0 (3)	C1—C2—H2	126.8
S2—C8—S1	125.5 (2)	C3—C2—H2	126.8
C12—C13—C14	117.1 (4)	C13—C12—C11	123.0 (4)
C12—C13—H13	121.4	C13—C12—H12	118.5
C14—C13—H13	121.4	C11—C12—H12	118.5
			
C7—N1—C9—C10	179.5 (4)	N2—C14—C13—C12	178.0 (4)
C8—N1—C9—C10	−3.1 (7)	N2—C7—C6—C5	−0.5 (7)
C7—N1—C9—C14	0.3 (4)	N1—C7—C6—C5	178.3 (4)
C8—N1—C9—C14	177.7 (4)	N2—C7—C6—S1	179.8 (4)
C13—C14—C9—C10	1.7 (6)	N1—C7—C6—S1	−1.4 (4)
N2—C14—C9—C10	−178.7 (3)	C8—S1—C6—C5	−179.0 (4)
C13—C14—C9—N1	−179.0 (3)	C8—S1—C6—C7	0.7 (3)
N2—C14—C9—N1	0.6 (4)	C7—C6—C5—C4	179.5 (4)
C8—N1—C7—N2	−179.2 (3)	S1—C6—C5—C4	−0.9 (6)
C9—N1—C7—N2	−1.2 (4)	N1—C9—C10—C11	−179.7 (4)
C8—N1—C7—C6	1.8 (5)	C14—C9—C10—C11	−0.7 (6)
C9—N1—C7—C6	179.7 (3)	C12—C11—C10—C9	0.5 (6)
N1—C7—N2—C14	1.5 (4)	C2—C3—C4—O1	0.7 (4)
C6—C7—N2—C14	−179.7 (4)	C2—C3—C4—C5	−179.5 (4)
C13—C14—N2—C7	178.2 (4)	C1—O1—C4—C3	−0.6 (4)
C9—C14—N2—C7	−1.3 (4)	C1—O1—C4—C5	179.5 (3)
C7—N1—C8—S2	177.2 (3)	C6—C5—C4—C3	175.6 (4)
C9—N1—C8—S2	0.0 (6)	C6—C5—C4—O1	−4.5 (6)
C7—N1—C8—S1	−1.2 (4)	C4—O1—C1—C2	0.2 (4)
C9—N1—C8—S1	−178.4 (3)	O1—C1—C2—C3	0.2 (5)
C6—S1—C8—N1	0.3 (3)	C4—C3—C2—C1	−0.5 (5)
C6—S1—C8—S2	−178.1 (3)	C14—C13—C12—C11	2.4 (6)
C9—C14—C13—C12	−2.5 (5)	C10—C11—C12—C13	−1.4 (6)

### Hydrogen-bond geometry (Å, º)

**Table:**

*D*—H···*A*	*D*—H	H···*A*	*D*···*A*	*D*—H···*A*
C10—H10···S2	0.93	2.94	3.476 (4)	119
C3—H3···N2^i^	0.93	2.62	3.474 (5)	154
C1—H1···S2^ii^	0.93	2.89	3.788 (4)	163

Symmetry codes: (i) −*x*+1/2, *y*+1/2, −*z*+3/2; (ii) −*x*, −*y*+1, −*z*+2.
