# Supplementary material for: Mol­ecular and crystal structure, Hirshfeld analysis and DFT investigation of 5-(furan-2-yl­methyl­idene)thia­zolo[3,4-a]benzimidazole-2-thione
Source: Acta Crystallogr E Crystallogr Commun. 2020 Nov 13;76(Pt 12):1832–6. doi: 10.1107/S2056989020015017 (PMC7784651; doi:10.1107/S2056989020015017)
Supplement: Supplementary file 4 [file e-76-01832-sup5.docx]

**Supplementary material**

**Molecular structure, HS analysis and DFT investigation of 5-(furan-2-yl-methylen)-thiazolo(3,4a) benzimidazole-2-thione**

Hafsa Khaldi,^a^ Ahmed Djafri,^b,c^ Youcef Megrouss,^c^ Nawel Khelloul,^c^ Abdelkader Chouaih ^c,^* and Ayada Djafri^a^

^a^ Laboratoire de Synthèse Organique Appliquée, Faculté des Sciences Exactes et Appliquées, Département de Chimie, Université Oran1, Algeria.

^b^ Centre de Recherche Scientifique et Technique en Analyses Physico-Chimiques (CRAPC), BP 384-Bou-Ismail-RP 42004, Tipaza, Algeria.

^c^ Laboratory of Technology and Solid Properties (LTPS), Abdelhamid Ibn Badis University of Mostaganem, 27000 Mostaganem, Algeria.

*Correspondence e-mail: abdelkader.chouaih@univ-mosta.dz, achouaih@gmail.com

**Selected bond lengths, bond angles and torsion angles for the non-hydrogen atoms, as determined by X-ray diffraction technique and DFT calculations**

| **Bond lengths (Å)** | **X-Ray** | **B3LYP/6–311G(d,p)** |
| --- | --- | --- |
| S1–C8 | 1.747(8) | 1.784 |
| S2–C8 | 1.603 (7) | 1.641 |
| O1–C1 | 1.356 (9) | 1.358 |
| C14–N2 | 1.401 (9) | 1.394 |
| N1– C8 | 1.400 (8) | 1.375 |
| C7 –N2 | 1.305 (8) | 1.299 |
| C11 –C12 | 1.379 (11) | 1.403 |
| C13– C12 | 1.375218 | 1.390 |
| C3 –C2 | 1.404618 | 1.422 |
| S1–C6 | 1.786(7) | 1.784 |
| N1 –C7 | 1.396 (9) | 1.403 |
| C6–C5 | 1.322218 | 1.354 |
| C5–C4 | 1.4262 | 1.422 |

| **Bond angles (°)** | **X-Ray** | **B3LYP/6–311G(d,p)** |
| --- | --- | --- |
| C8– S1– C6 | 95.1(3) | 93.463 |
| C7– N1– C9 | 107.1(6) | 106.201 |
| S2– C8– S1 | 125.8(4) | 123.762 |
| C1– C2– C3 | 106.3(7) | 106.231 |
| C1– O1– C4 | 104.3(6) | 107.319 |
| N1– C8– S1 | 107.4(5) | 108.179 |
| C6– C5– C4 | 128.7(7) | 128.304 |
| C3– C4– C5 | 134.0(7) | 131.725 |
| C11 –C12– C13 | 123.4(7) | 121.440 |

| **Torsion angles (°)** | **X-Ray** | **B3LYP/6–311G(d,p)** |
| --- | --- | --- |
| C7 – N1 – C8 –S2 | 177.4(6) | 179.93 |
| C6 –C5 – C4 – O1 | -4.3(11) | 0.012 |
| C9 –N1 – C8 – S2 | 0.2(13) | 0.124 |
| S1 – C6 – C5 –C4 | -1.0(12) | -0.0006 |
| C6 – C5 – C4 –C3 | 176.1(9) | 179.99 |
